# Supplementary material for: Complexome profiling of the Chlamydomonas psb28 mutant reveals TEF5 as an early PSII assembly factor
Source: Plant Cell. 2025 Mar 17;37(6):koaf055. doi: 10.1093/plcell/koaf055 (PMC12164590; doi:10.1093/plcell/koaf055)
Supplement: koaf055_Supplementary_Data [file koaf055_supplementary_data.zip › psb28_interactive.zip!/plots/intensity/qualityPlots/peptides/_Cre12.g551900.t1.2.html]

### Cre12.g551900.t1.2

Trivial name:   
SeqCoverage [median]: 32.10  
Mass: 15.85 kDa  
  
 

| Sequence | MissedCleavage | Mass [Da] | wtIntensity | muIntensity | Score |
| --- | --- | --- | --- | --- | --- |
| AFMPQSIQDVQK | 0 | 1390.69 | 424.19 | 0.00 | 75.65 |
| AVENMIEDLQK | 0 | 1288.63 | 0.00 | 1092.57 | 115.23 |
| LKADMLAALR | 1 | 1100.64 | 0.00 | 602.50 | 43.30 |
| VIQQALGDFQER | 0 | 1402.72 | 450.24 | 6392.80 | 132.88 |
|  |  |  | 874.4 | 8087.9 |  |
